# Achiasmy: Male Fruit Flies Are Not Ready to Mix

**DOI:** 10.3389/fcell.2016.00075

**Published:** 2016-07-19

**Authors:** Alphy John, Kavya Vinayan, Jishy Varghese

**Affiliations:** Drosophila Research in Energy and Metabolism Lab, School of Biology, Indian Institute of Science Education and ResearchThiruvananthapuram, India

**Keywords:** Drosophila meiotic recombination, crossover, homologous recombination, achiasmy

## Abstract

Maintenance of the chromosomal copy number over generations and recombination between homologous chromosomes are hallmarks of meiotic cell division. This genetic exchange that take place during gamete formation leads to genetic diversity, the main driving force behind natural selection. Formation of chiasmata, the physical link between homologous chromosomes during meiosis, is a requisite for recombination. In addition, chiasmata also aid in proper segregation of homologous chromosomes and has a major impact on reproductive fitness. Given these facts it is intriguing that many insect species have forgone the need for genetic exchange between homologous chromosomes during meiosis. Geneticists for several decades knew that meiotic crossover and recombination is absent in *Drosophila* males and some female lepidopterans, a condition termed achiasmy. However, a good understanding of the mechanisms that cause achiasmy and the evolutionary benefits of achiasmy is currently lacking. In this article we will discuss possible genetic and molecular basis of achiasmy in male *Drosophila*.

## Introduction

Meiotic cell division, an essential step in sexual reproduction, helps in the segregation of homologous chromosomes and sister chromatids. In addition, a crucial task for meiotic cell division is the maintenance of recombination mediated genetic variability (Hunter, 2015). A “standard meiotic script” and maintenance of high-fidelity during chromosomal segregation is well conserved among eukaryotes (Nicklas, 1997; McKee, 2004). Mis-segregation of homologs during meiosis leads to aneuploidy which causes lethality or genetic disorders in the offsprings. Aneuploidy is a major cause for approximately one-third of spontaneous miscarriages in humans, developmental disabilities, and mental retardation (Hassold et al., 2007).

The greatest advantage of sexual reproduction, which otherwise is a bottle neck due to the complexities involved, is meiotic recombination. Recombination yields newer combinations of alleles, which helps in the genetic adaptability of the organism (Carvalho, 2003). The adverse effects of the absence of meiotic recombination is clear from Steinmann's analysis of genes in *Drosophila miranda* “neo-Y chromosome,” which resulted from the fusion of an autosome to the Y-chromosome estimated to have happened a million years ago (Bachtrog, 2005). The genes on the attached autosome (neo-Y) underwent degeneration due to the lack of recombination during meiosis in *Drosophila* males, while its homolog neo-X remained intact in females due to the existence of recombination. Extending this observation, Bachtrog et al. showed that deleterious mutations accumulate on a non-recombining chromosome (Bachtrog and Charlesworth, 2000, 2002). The human Y chromosome has been shown to reduce errors in the coding regions by having a self-recombination mechanism (Rozen et al., 2003; Skaletsky et al., 2003). An added advantage of meiotic recombination is that the chiasmata formation during crossover helps in proper alignment and segregation of chromosomes (Carpenter, 1994). Given the benefits, it is confounding that meiotic recombination is absent in some species.

**Figure 1:**
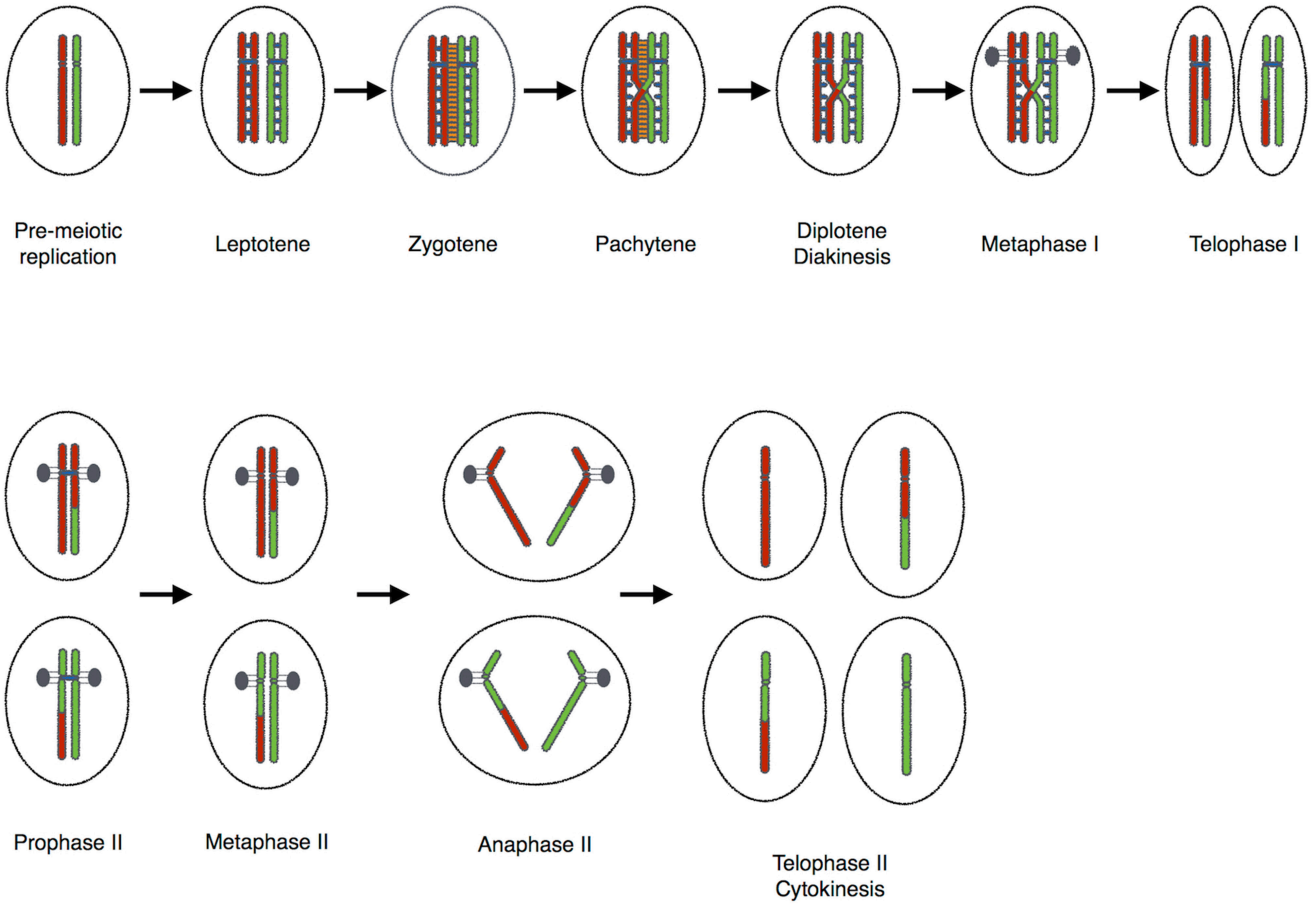
**The “standard meiotic script”**.

Loss of meiotic recombination results in aneuploidy in plants, but with less deleterious consequences than in animals. This is a boon for plant breeders and farmers due to the obvious advantages (Caryl et al., 2003). Heterochiasmy, the dimorphism in meiotic recombination rates between sexes is seen in various divergent species. Several hypotheses have been proposed to explain the evolution of heterochiasmy (Lenormand, 2003; Lenormand and Dutheil, 2005). Achiasmy a form of heterochiasmy, where males or females of a species completely lack meiotic recombination, occurs frequently in Dipterans and in several orders of Lepidopterans. According to the Haldane-Huxley rule, it is the heterogametic sex (XY or WZ) that shows achiasmic meiosis. Morgan (1910) was the first to describe achiasmy in *Drosophila* males (Morgan, 1910). However, *Drosophila* females, like the majority of sexually reproducing organisms, generate crossovers between homologous chromosomes to direct segregation at the first meiotic division (Lindsley and Sandler, 1977; Puro and Nokkala, 1977; Lin et al., 1981; Orr-Weaver, 1995; Lichten, 2001; McKim et al., 2002; Figure 1). During meiosis the male germ-line cells of fruit flies undergo homolog pairing of chromosomes creating bivalents that can be sequestered to unique territories inside the Prophase nucleus (Hawley, 2002). However, no genetic exchange occurs during this process. Interestingly, there are rare reports of spontaneous meiotic recombination in male *Drosophila melanogaster* (Hiraizumi, 1971).

Achiasmy in male fruit flies arose at least tens of million years ago as it is a common trait in the *Drosophila* clade, this raises several interesting questions:

- How can an evolutionarily conserved process like meiotic recombination be excluded in a sex-specific manner?
- How can a trait that helped in laying the foundation for natural selection get erased completely from one sex?
- In spite of the risks of accumulation of deleterious mutations how does heterochiasmic species benefit from forgoing meiotic recombination?

In addition to several invertebrates many vertebrates exhibit lower recombination frequency in the heterogametic sex, which is usually male. However, it has so far been hard to establish if the higher recombining sex would compensate for the low recombination rates in the other sex. The fact that achiasmy is observed in one sex (mostly in heterogametic sex) argues that there could be compensation of recombination rates in the other sex. In mice, the female sex chromosomes have more chiasmata for their length than the autosomes, this is probably to compensate for the lower levels of recombination in males (Burt et al., 1991). However, there is no direct evidence for compensation in the recombining sex.

We tried to figure out whether compensation of recombination rates exists in heterochiasmic species by comparing closely related chiasmic and achiasmic species. Among Drosophilids male recombination have been recorded from *Drosophila ananassae* and *Drosophila willistoni*, species closely related to achiasmic *D. melanogaster*. Comparison of recombination rates in autosomes of females of *D. ananassae* and *D. melanogaster* do not show significant differences. The only exception is the X-chromosome in *D. melanogaster*, which has lower recombination rates when compared to autosomes and to *D. ananassae* (Caceres et al., 1999). After taking the above fact in to consideration it could be concluded that at least in *Drosophila melanogaster* there is no obvious compensation of recombination rates in chiasmate females. Also, using simple mathematical calculations we found that the absence of recombination does not lead to an overall effect on genetic variability (see Supplementary Material for details).

One prevailing hypothesis regarding achiasmy in male *Drosophila* is the “Lazy Male hypothesis.” According to this hypothesis, the non-recombining males that has diverted the recombination task entirely to females could be more fit in comparison. This might allow the non-recombining sperms gain increased fitness resulting from the conservation of energy due to lack of recombination events and result in enhanced fecundity. Another suggestion based on Haldane's views is that the loss of recombination in males could have evolved as a mechanism for preventing recombination of the male sex-chromosome.

Our attempt in this article is not to explain why recombination is absent in male fruit flies, we will try to elaborate on the possible genetic and molecular reasons behind achiasmy in male *Drosophila* here. We propose that the absence of meiotic recombination in males is due to the absence of key recombination factors or is due to the presence of negative factors that prevent recombination from occurring during spermatogenesis.

## How different is *Drosophila* male meiosis?

Though the basic archetype of meiosis is met in *Drosophila* male meiosis, the chromosomal pairing events do not facilitate genetic exchange, but create bivalents that are assigned with discrete territories in the Prophase nucleus (Fabian and Brill, 2012). Near the apical end of the testis, are the cells that undergo Meiosis I, the spermatocytes. The Prophase I stage in *Drosophila* males show non-conventional phenotypes as the chromosomes of male fruit flies are indistinguishable from G2 phase and lack structural features of a traditional Prophase I. A trilobular nucleus is visible at this stage, corresponding to three major bivalents in the nucleus. Prominent axes normally decorated with cohesins and other lateral element proteins are missing from the Prophase I chromosomes. Bivalents are also not attached by classical chiasmata. Another deviation from the “standard script” is that the homologs enter the meiotic cycle already paired, abolishing the need for homolog search. Since there is no recombination during Drosophila male meiosis the synaptonemal complex is undetectable. However, the pairing sites on the homologs are bound by specific protein complexes that ensure the legitimate segregation of the homologs (Ault et al., 1982; Ault and Rieder, 1994; Vazquez et al., 2002; McKee et al., 2012). To find the possible mechanisms behind achisamy in male *Drosophila*, we compared the expression of genes between adult ovary and testis using GEO2R (Barrett et al., 2013). This analysis showed downregulation of several recombination specific genes in testis in comparison to ovary. The genes which show differential expression between sexes are listed down in Table 1, and the functions of some interesting candidate genes are discussed in detail.

**Table 1:** **Genes playing a role during meiosis that show differential expression between testis and ovary (Data acquired from GEO Dataset GSE7763, contributed by Chintapalli VR, Wang J, and Dow JA available at flyatlas.org; Chintapalli et al., 2007)**.

**No**.	**Name**	**Symbol**	**Biological process**	**logFC**
1	*deadhead*	*dhd*	female meiosis, cellular response to DNA damage stimulus	−6.43
2	*PTIP associated 1*	*Pa1*	histone H3-K4 methylation	−5.21
3	*Protein phosphatase 2B at 14D*	*Pp2B-14D*	female meiosis	−4.52
4	*centrosomin*	*cnn*	female meiosis chromosome segregation	−4.48
5	*teflon*	*tef*	male meiosis	−4.34
6	*crossover suppressor on 2 of Manheim*	*c(2)M*	meiotic nuclear division, reciprocal meiotic recombination, resolution of meiotic recombination intermediates	−3.77
7	–	*pds5*	karyosome formation, sister chromatid cohesion, chromosome segregation	−3.45
8	*modifier of mdg4*	*mnm*	regulation of chromatin assembly or disassembly, male meiosis I, male meiosis chromosome segregation	−3.32
9	*grapes*	*grp*	DNA damage checkpoint, female meiosis chromosome segregation, spindle assembly	−3.3
10	*out at first*	*oaf*	female meiosis chromosome segregation	−3.28
11	*Breast cancer 2, early onset homolog*	*Brca2*	double-strand break repair via homologous recombination, meiotic recombination checkpoint	−3.24
12	*twine*	*twe*	male meiosis, spermatogenesis, spindle assembly involved in female meiosis, spindle assembly involved in male meiosis	−3.23
13	*recombination-defective*	*rec*	reciprocal meiotic recombination, DNA replication	−3.13
14	*spindle B*	*spn-B*	germarium-derived oocyte fate determination, meiotic nuclear division, reciprocal meiotic recombination	−3.06
15	*crossover suppressor on 3 of Gowen*	*c(3)G*	reciprocal meiotic recombination	−2.71
16	*Myt1*	*Myt1*	female meiosis, male meiosis, meiotic nuclear division	−2.64
17	*Minichromosome maintenance 10*	*Mcm10*	female meiosis chromosome segregation	−2.49
18	*Calcineurin B2*	*CanB2*	meiotic nuclear division	−2.48
19	*Topoisomerase 2*	*Top2*	meiotic nuclear division, mitotic recombination, mitotic sister chromatid segregation	−2.41
20	*Gamma-tubulin ring protein 84*	*Grip84*	meiotic nuclear division, spermatogenesis	−2.31
21	*Grip128*	*Grip128*	spindle assembly involved in female meiosisII, male meiosis cytokinesis	−2.11
22	*spindle A*	*spn-A*	DNA recombination, DNA repair, oogenesis	−1.58
23	*meiotic W68*	*mei-W68*	meiotic DNA DSB formation, meiotic recombination nodule assembly, oogenesis	−0.02
24	*meiotic from via Salaria 332*	*mei-S332*	sister chromatid cohesion	0.73
25	*meiotic P22*	*mei-P22*	reciprocal meiotic recombination, meiotic DNA double-strand break formation	0.74
26	*Bloom syndrome helicase ortholog*	*Blm*	cellular response to DNA damage stimulus, reciprocal meiotic recombination	1.11
27	–	*PI31*	male meiosis	2.12
28	*meiotic 217 and 218*	*mei-218/217*	female meiosis chromosome segregation	2.24
29	*mushroom body defect*	*mud*	spindle assembly involved in female meiosisII	2.99
30	*achintya*	*achi*	spermatogenesis	3.12
31	*orientation disruptor*	*ord*	gamete generation, meiotic nuclear division, sister chromatid cohesion, chromosome segregation, female meiosis sister chromatid cohesion	3.56
32	*corolla*	–	synaptonemal complex assembly, female meiotic division, meiotic DNA double-strand break processing	3.59
33	*Stromalin-2 (snm)*	*snm*	male meiosis	4.15
34	*corona*	*cona*	synaptonemal complex assembly	6.09
35	–	*klhl10*	sperm individualization	8.48
36	*Rac GTPase activating protein at 84C*	*RacGAP84C*	spermatogenesis	8.75
37	*Heterochromatin protein 6*	*HP6*	female meiosis	8.87
38	*sungrazer*	*sunz*	male meiosis	9.8
39	*walker cup*	*wa-cup*	male meiosis	10.05
40	*Skadu*	*Skadu*	chromosome organization	11.43

*Analyzed using GEO 2R. logFC is the log fold change in gene expression in male testis compared to the female ovary. Negative value indicates lower expression in testis compared to the ovary*.

## Chiasmata substitutes in males: MNM, SNM, and TEF

Accurate homolog segregation depends on the pairing of homologs that form bivalents that interact with the meiotic spindle as a unit (Roeder, 1990). During meiosis, *Drosophila* females utilize chiasmata to pair up their three major homologous chromosomes (McKim et al., 1998). By contrast, even in the absence of chiasmata or synaptonemal complex formation and recombination, all the four chromosome pairs form stable bivalents in males (Ren et al., 1997).

The 200–250 copies of rRNA genes share homology in the *Drosophila* X and Y chromosomes. Stromalin in Meiosis (SNM) and Modifier of Mdg4 in Meiosis (MNM) are present in the X–Y pairing sites and are required for stable homolog pairing and segregation in male and not for female meiosis (Thomas et al., 2005). On the contrary, recruitment of SNM and MNM to autosomes depends on another protein TEFLON (TEF). Flies that lack *tef*, *snm*, and *mnm* show phenotypes during male meiosis, but not in female meiotic cells (Thomas et al., 2005). Each autosomal homolog is seen in a common territory till the late-Prophase I, SNM and MNM localize to these homolog territories and at pro-Metaphase I they start condensing into well aligned bivalents. This is lost in the *mnm* and *snm* mutants, which suggests that they help in bringing the homologs together into a common territory. Based on the FlyAtlas data *snm* transcript levels are more than 20-fold higher in testis compared to ovary. On the contrary, *mnm* and *tef* transcripts are very low in testis, though they are essential for the formation of chromosomal territories during male meiosis. One possible explanation for the lack of meiotic recombination in *Drosophila* males is that high levels of SNM might prevent recombination factors from acting. This can be addressed by expressing SNM in female germ cells and check effects on meiotic recombination. A change in the expression or binding of the recombination initiators in these females will answer a part of our query.

## DNA machetes and synaptonemal complex components

In most organisms, meiosis proceeds with synaptonemal complex (SC) formation in a DSB-dependent fashion. Quite contrary to this, SC formation precedes DSB formation and recombination events, and is a necessary step in Drosophila females, evident from mutant analyses (Jang et al., 2003; Mehrotra and McKim, 2006). A marker for SC showed that SC is present before DSB protein MEI-P22 foci appear on the chromosomes (Liu et al., 2002). Several components of SC in Drosophila females have been identified. C(3)G constitutes the transverse filaments (TFs) of SC (Page and Hawley, 2001). The central elements (CEs) are bound by the N-termini of C(3)G homodimers, while the C-termini help the TFs to form connections via axial/lateral elements (AEs/LEs). *c(3)G* mutants lack both SC formation and MEI-P22 foci, suggesting that DSB formation is dependent on SC formation in Drosophila females. In addition, these mutants completely lack genetic exchange during meiosis (Jeffress et al., 2007; Page et al., 2008). C(2)M helps the TFs to bind to the chromosomes and co-localizes with C(3)G. Corona (Cona) is a component of AEs, that is found to co-localize with C(3)G and is essential for the polymerization of C(3)G monomers. Recombination frequency in *corona* mutant females is found to be 50-to-200 fold lower. Another component of AEs is Corolla, which interacts with Cona, to stabilize the SC structure. *corolla* mutants show increased non-disjunction compared to the wildtype females. *c(2)M* mutants also show reduced meiotic crossover frequency (Manheim and McKim, 2003). Though levels of *corolla* and *corona* are high in testis, levels of *c(3)G* and *c(2)M* transcripts are very low in male testis, and could be key contributors to the lack of meiotic recombination (Anderson et al., 2005).

DSBs have been shown to be adept in initiating recombination in meiotic cells of Baker's yeast. Experiments in *Drosophila* females show that DSBs can act as recombination initiators during meiotic division. *mei-W68* encodes the Baker's yeast recombinase *spo11* homolog, which is required for the DSB initiation during meiotic recombination in *Drosophila* females (McKim and Hayashi-Hagihara, 1998). *mei-P22* produces another factor for DSB formation, and have mutant phenotypes similar to *mei-W68* (Liu et al., 2002). MEI-P22 foci is present for a short time during early meiotic prophase. However, our analysis do not reveal a huge difference in the levels of *mei-P22* and *mei-W68* levels in testis compared to ovary. Lack of expression of MEI-P22 and MEI-W68 proteins due to post-transcriptional regulation or lack of activation of these DSB proteins by post-transcriptional modifications could be responsible for achiasmy in male *Drosophila*. As synaptonemal complex formation is a pre-requisite for MEI-P22 association with the chromosomes in *Drosophila* as seen from the *c(3)G* mutants, it is not hard to assume that the MEI-P22 and MEI-W68 proteins are unable to generate DSBs in male *Drosophila*.

## *Drosophila* RecA homolog: SpnA

The key player of recombination in prokaryotes is RecA, which catalyses the pairing and strand invasion between homologous DNA strands, during both DNA repair and crossover recombination (Shinohara and Shinohara, 2004). RAD51 and DMC1, two RecA like proteins, in yeast are required for meiotic recombination. In *Drosophila*, SpnA (DmRAD51), shares strong sequence similarity with RAD51 protein of yeast, chicken, mouse, and human. *spnA* mutant females show a significant elevation in the frequency of DSBs during meiotic recombination (Staeva-Vieira et al., 2003) and also show single-strand annealing (SSA) repair than DSB repair through crossover and recombination during meiosis (Yoo and McKee, 2004). The levels of *spnA* transcript is significantly lower in testis during spermatogenesis and could be a limiting factor responsible for the absence of meiotic cross-over in male *Drosophila*.

## BLM helicase and MCM proteins

Unlike meiotic crossovers that are beneficial, mitotic crossovers can lead to the loss of heterozygosity, possibly increasing the chances of tumorigenesis (Andersen and Sekelsky, 2010; Kohl and Sekelsky, 2013). As a safeguard mechanism mitotic crossovers are prevented by anti-crossover proteins like the helicase BLM, which unwinds the recombination intermediates during mitosis to generate non-crossover products, across metazoans. Generation of meiotic crossovers in most eukaryotes requires the removal of the anti-crossover proteins by Msh4–Msh5 complex. *Drosophila* lost *msh4* and *msh5* genes, which is functionally replaced by Mini-Chromosome Maintenance (MCM) complex proteins. Mutants of *rec, mei-217* and *mei-218*, genes which encode for *Drosophila* MCM complex, show reduced female meiotic crossovers, which can be rescued by the removal of *blm* gene (Kohl et al., 2012). Transcript levels of *blm* is 2-fold higher in male testis; this and the MCM loss of function phenotypes in females suggest a role for BLM helicase in inhibiting meiotic crossovers in male fruit flies. However, while *mei-217* and *mei-218* transcript levels are significantly high in the testis, *rec* transcript levels are very low. An increased rate of non-crossover recombination was observed in *rec* mutant females, about 2-fold, not surprising as it is an MCM complex protein (Carpenter, 1975, 1989; Bojko, 1989; Matsubayashi and Yamamoto, 2003). As *mei-217* and *mei-218* expression is enhanced in testis according to the microarray data [GEO: Dataset GSE7763], it is possible that the low levels of *rec* transcripts in *Drosophila* males leads to the lack of inhibition of BLM anti-crossover proteins. *Drosophila* males mutant for *blm* gene could provide an answer to this. Overexpression of REC protein in testis would also reveal whether lack of functional MCM complex is responsible for lack of meiotic recombination in males.

## Other interesting genes

We have not covered all the possible factors that could be responsible for achiasmy in male Drosophila here due to various limitations. Other meiotic genes like *grapes* (*grp*) and *out at first* (*oaf*), both needed for chromosome segregation (Dobie et al., 2001); *deadhead* (*dhd*), a thioredoxin homolog (Salz et al., 1994) and *topoisomerase 2* (*top2*; Hughes and Hawley, 2014); show very low expression in the male testis. These genes are enriched in the germ line progenitors, the pole cells, during embryogenesis (Mukai et al., 2006). It is possible that some of these gene products could also play a prominent role in the absence of meiotic cross over in *Drosophila* males.

## Looking forward

Generation of haploid gametes from diploid precursors by meiosis is a crucial step during sexual reproduction. During this process genetic exchange occurs, introducing variability in the population by mixing genotypes and also safeguards the segregation of the homologous chromosome pairs. Recent studies have thrown light on the possible mechanisms by which homologs are efficiently segregated in the absence of chiasmata and cross-over recombination during *Drosophila* spermatogenesis. What are the mechanisms that control sex-specific shutdown of meiotic recombination in one sex of several species like *Drosophila melanogaster*? And, why there is sex-specific shutdown of meiotic recombination in some species? A number of genes important for steps of crossover and meiotic recombination show differential expression when microarray data for gene expression between testis and ovary of fruit flies were compared. There are significant changes in the levels of transcripts of genes that encode for recombination factors in the testis compared to the ovary. The precise molecular pathways that regulate the expression of these recombination factors still remain to be discovered. We speculate that the absence or inactivation of key players of genetic recombination during spermatogenesis might be the cause for achiasmy in *Drosophila* males. This can be tested by attempting to restore meiotic recombination in male flies by the germ-line specific expression of these factors. As achiasmy could be a result of absence of multiple recombination-specific factors this may not be easy. Another factor to consider, which is a limitation of our analysis, is the lack of proteomics data or information related to post-translational modification of various recombination factors in testis and ovary. The discovery of a strain isolated from the wild that exhibit recombination in males is suggestive of the fact that few factors/genes could also be responsible for the lack of this genetic event in male *Drosophila* (Hiraizumi, 1971). The question remains whether there is a master regulator of meiotic recombination in male *Drosophila*. The genes that show very high expression in testis could be tested for this role. Research in this direction could possibly unravel reasons for fertility defects and disorders associated with aneuploidy.

## Author contributions

AJ, KV, and JV conceptualized the idea behind this manuscript. AJ and KV performed the bioinformatics analysis and wrote the initial draft of this manuscript. JV generated the final draft of this manuscript. JV also generated the figures and tables for this manuscript.

## Funding

AJ and KV were supported by the DST-Inspire Scholarship program while working toward this manuscript. JV was supported by DST-SERB Ramanujan Fellowship while working toward this manuscript. JV would like to thank IISER TVM and Ministry of Human Resources and Development, India for the generous financial support to his laboratory.

### Conflict of interest statement

The authors declare that the research was conducted in the absence of any commercial or financial relationships that could be construed as a potential conflict of interest.
