# Supplementary material for: Achiasmy: Male Fruit Flies Are Not Ready to Mix
Source: Front Cell Dev Biol. 2016 Jul 19;4:75. doi: 10.3389/fcell.2016.00075 (PMC4949207; doi:10.3389/fcell.2016.00075)
Supplement: Supplementary file 1 [file Presentation1.PDF]

Supplementary material:

Mathematical explanation:

We made some assumptions to simplify the calculations:
1) Rate of recombination is constant
2) Only one recombination happens between two genes

Let's consider two heterozygous parents: AaBb and AaBb
Assume that one recombination event occur between genes A and B during meiosis then the alleles are A, a and B, b and the gametes will be Ab, AB, aB and ab

1) <u>If recombination occurs at an equal rate in both sexes: (Ideal case)</u>

Gametes from female: Ab, AB, bA and ab
Gametes from male: Ab, AB, bA and ab
Among 16 possible progenies, 2 will be of parental type (underlined ones) and rest will be recombinants.

	**AB**	**Ab**	**aB**	**Ab**
**AB**	<u>AABB</u>	AABb	AaBB	AaBb
**Ab**	AABb	AAbb	AaBb	Aabb
**aB**	AaBB	AaBb	aaBB	aaBb
**ab**	AaBb	Aabb	aaBb	<u>Aabb</u>

So the ratio of recombinant progeny will be 14/16 = 7/8

2) <u>In case of achiasmy in one sex (male):</u>

Gametes from female: Ab, AB, bA and ab
Gametes from male: AB and ab

	**AB**	**Ab**	**aB**	**Ab**
**AB**	<u>AABB</u>	AABb	AaBB	AaBb
**ab**	AaBb	Aabb	aaBb	<u>Aabb</u>

So the ratio of recombinant progeny is: = 6/8

The difference in frequency of recombinant progeny between ideal (recombination in both sexes) and achiasmy is: (7/8) - (6/8) = 1/8

If we consider recombination between 3 genes: A, B and C and the parents are heterozygous for these genes AaBbCc

Then, The difference in frequency of recombinant progenies between ideal (recombination in both sexes) and achiasmy is: (31/32) - (14/16) = 3/32

If we extrapolate this to '***m***' genes, the difference in frequency of recombinant progenies between ideal (recombination in both sexes) and achiasmy = $[2^{(m-1)}-1)/(2^{(2m-1)}]$

As the number '***m***' increases, the difference in genetic variability between ideal and achiasmy conditions becomes negligible. This suggests that, shutting down recombination in one sex does not affect the genetic diversity in a population. Hence, the male flies might be directing their energy for producing more sperms rather than wasting it for an energy consuming process like recombination, to increase the chance of viable fertilizations.
